# Synthetic Data Generation via Generative Adversarial Networks in Healthcare: A Systematic Review of Image- and Signal-Based Studies

**DOI:** 10.1109/OJEMB.2024.3508472

**Published:** 2024-11-28

**Authors:** Muhammed Halil Akpinar, Abdulkadir Sengur, Massimo Salvi, Silvia Seoni, Oliver Faust, Hasan Mir, Filippo Molinari, U. Rajendra Acharya

**Affiliations:** Vocational School of Technical SciencesIstanbul University-Cerrahpasa 532719 34320 Istanbul Türkiye; Technology FacultyFirat University 23119 Elazig Türkiye; Department of Electronics and TelecommunicationsPolitecnico di Torino 19032 10129 Turin Italy; Anglia Ruskin University Cambridge Campus 150697 CB1 1PT Cambridge U.K.; American University of Sharjah Sharjah 26666 UAE; University of Southern Queensland 213175 Toowoomba QLD 4300 Australia

**Keywords:** Generative adversarial networks (GANs), medical imaging, data generation, signal simulation, deep learning

## Abstract

Generative Adversarial Networks (GANs) have emerged as a powerful tool in artificial intelligence, particularly for unsupervised learning. This systematic review analyzes GAN applications in healthcare, focusing on image and signal-based studies across various clinical domains. Following Preferred Reporting Items for Systematic reviews and Meta-Analyses (PRISMA) guidelines, we reviewed 72 relevant journal articles. Our findings reveal that magnetic resonance imaging (MRI) and electrocardiogram (ECG) signal acquisition techniques were most utilized, with brain studies (22%), cardiology (18%), cancer (15%), ophthalmology (12%), and lung studies (10%) being the most researched areas. We discuss key GAN architectures, including cGAN (31%) and CycleGAN (18%), along with datasets, evaluation metrics, and performance outcomes. The review highlights promising data augmentation, anonymization, and multi-task learning results. We identify current limitations, such as the lack of standardized metrics and direct comparisons, and propose future directions, including the development of no-reference metrics, immersive simulation scenarios, and enhanced interpretability.

## I. Introduction

Artificial intelligence (AI) has revolutionized healthcare, enabling more accurate diagnoses, personalized treatments, and efficient clinical workflows. However, AI model development and validation in medicine are often limited by the scarcity of high-quality, representative medical data due to privacy concerns and high acquisition costs [1].

**Fig. 1.**
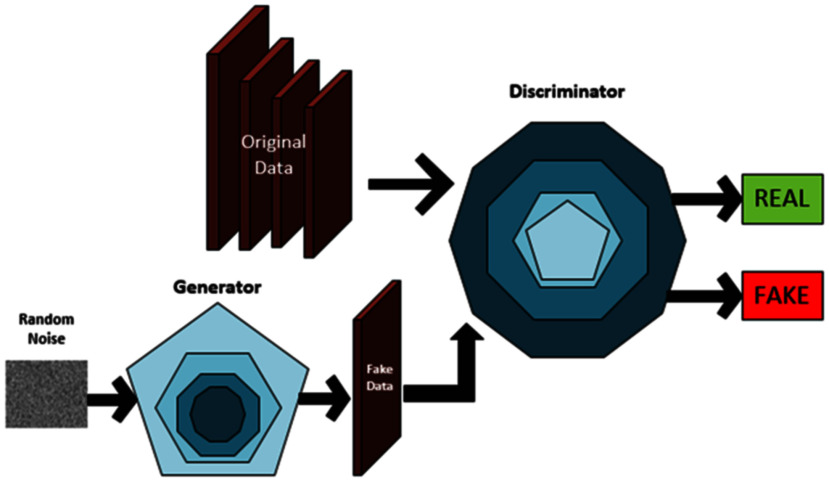
Diagram of a generative adversarial network (GAN). The generator creates synthetic medical data from random noise, while the discriminator learns to distinguish between real and synthetic samples.

To address these limitations, Goodfellow et al. [2] introduced Generative Adversarial Networks (GAN), a novel class of deep learning models. GANs learn from real data distributions to generate realistic synthetic data, increasing data availability. The GAN architecture consists of two competing neural networks: the generator, which creates synthetic data, and the discriminator, which distinguishes between real and synthetic data. Through an adversarial training process, the generator produces increasingly realistic data to fool the discriminator. Fig. 1 displays an overview block diagram of the GAN model.

Since its invention, GAN has been incorporated into solutions for a wide range of medical problems, from creating synthetic medical images [3] to generating electronic health records [4] and physiological signals [5]. GANs can improve AI model performance by augmenting existing datasets with synthetic data, facilitating data sharing while maintaining patient privacy [6], and enhancing applications such as image super-resolution [7], and cross-modality image synthesis [8].

Despite growing interest, challenges persist in medical applications of GANs. Effective use requires rigorous data handling methodologies and quality assessment to ensure diversity and clinical relevance of synthetic data [9]. The lack of standardized metrics and benchmarks complicates the comparison of different GAN models and the assessment of their real-world impact [10].

This systematic review aims to provide a comprehensive overview of GAN-based synthetic data generation methods in healthcare, focusing on image and signal-based studies across various clinical domains. The main objectives are:

• Identification of key GAN architectures and training strategies that are used for medical image and signal synthesis.
• Analysis of datasets, evaluation metrics, and performance of reported GAN models across different clinical applications.
• Discussion of challenges, limitations, and future directions of the GAN model in healthcare.

### A. Search strategy

During our literature survey, we found several review papers exploring GAN-related applications in the clinical domain. Most focused on specific aspects, such as medical images [11], cellular imaging [12], or data augmentation. In contrast, our review offers a comprehensive overview of GAN models for data generation across various medical domains and modalities.

Previous reviews have addressed GANs in medical, such as cancer imaging [11], molecular imaging [12], medical image classification and segmentation [13], time series signals [14], and data augmentation for electrocardiogram (ECG) signals [15]. However, these reviews present limited analyses of specific clinical areas, modalities, or time periods, which hinders their ability to capture the full potential and diverse applications of GANs in medical imaging and signal analysis.

## II. Background

### A. GAN Components and Objective Function

The GAN framework consists of two main components: the generator and the discriminator. The generator $G( {z,\ {{\theta }_g}} )$ learns the data distribution ${{p}_g}$ over data x, taking random noise z from distribution ${{p}_z}$ as input to generate synthetic samples. The discriminator $D( {x,\ {{\theta }_d}} )$ is a binary classifier that estimates the probability that given data x is real rather than synthetic [16]. The GAN training process is framed as a minimax game with the following value function:

\begin{align*}
{\min}_{G}{\max}_{D}V\left( {G,\ D} \right) =& \ {{\mathbb{E}}_{x\sim {{p}_{data}}\left( x \right)}}\left[ {logD\left( x \right)} \right] \\
&+ \ {{\mathbb{E}}_{z\sim {{p}_z}\left( z \right)}}\left[ {\log \left( {1 - D\left( {G(z} \right)} \right))} \right] \tag{1}
\end{align*}

The discriminator aims to maximize $V( {G,\ D} )$ to correctly label real and generated data, while the generator aims to minimize $V( {G,\ D} )$ to produce data that the discriminator will classify as real. Training proceeds in alternating steps, updating *D* and *G* in turn [2].

### D. GAN Variations

The literature proposes various GAN architectures for a wide range of tasks, such as synthetic data generation, data enhancement, and transformations.

Conditional GAN (cGAN), introduced by Mirza and Osindero [17], incorporates additional conditions (e.g., labels or features) to guide the generator's output, enabling controlled generation of data with specific characteristics. Progressive Growing of GAN (PGGAN), proposed by Karras et al. [18], gradually increases the resolution of generated images by progressively adding layers to both generator and discriminator, resulting in more stable training and higher quality outputs.

Pixel-to-pixel GAN (Pix2Pix), developed by Isola et al. [19], uses paired datasets for image-to-image translation tasks, where the generator transforms input images into corresponding outputs. Cycle GAN, introduced by Zhu et al. [20], enables unpaired image-to-image translation by incorporating cycle consistency loss, allowing transformations between domains without paired examples. Style GAN, presented by Karras et al. [21], introduces a style-based generator architecture, offering fine-grained control over generated image attributes at different scales.

Deep Convolutional GAN (DCGAN), proposed by Radford et al. [22], integrates convolutional neural networks into the GAN framework, improving stability and quality of generated images. These variations have expanded the capabilities of GANs, enabling more diverse and specialized applications in medical imaging and signal analysis.

## III. Method

This systematic review follows the preferred reporting items for systematic reviews and meta-analyses (PRISMA) guidelines [23] to investigate GAN use in healthcare, focusing on articles published until February 1, 2024. A literature search was conducted in October 2023 across IEEE Xplore, PubMed, Web of Science, and Scopus databases using a Boolean approach, combining various keywords such as "health" OR "medical" OR "patient" AND "image generation" OR "image synthesis" OR "synthetic image" OR "signal generation" OR "signal synthesis" OR "synthetic signal" OR "ECG" OR "EEG" OR "EMG" OR "EOG" OR "PPG" AND "generative adversarial network*" OR "GAN*". The initial search yielded 1131 articles, screened for duplicates and relevance, resulting in 903 articles. Further review excluded non-journal sources, non-English publications, and literature reviews (n = 524).

**Fig. 2.**
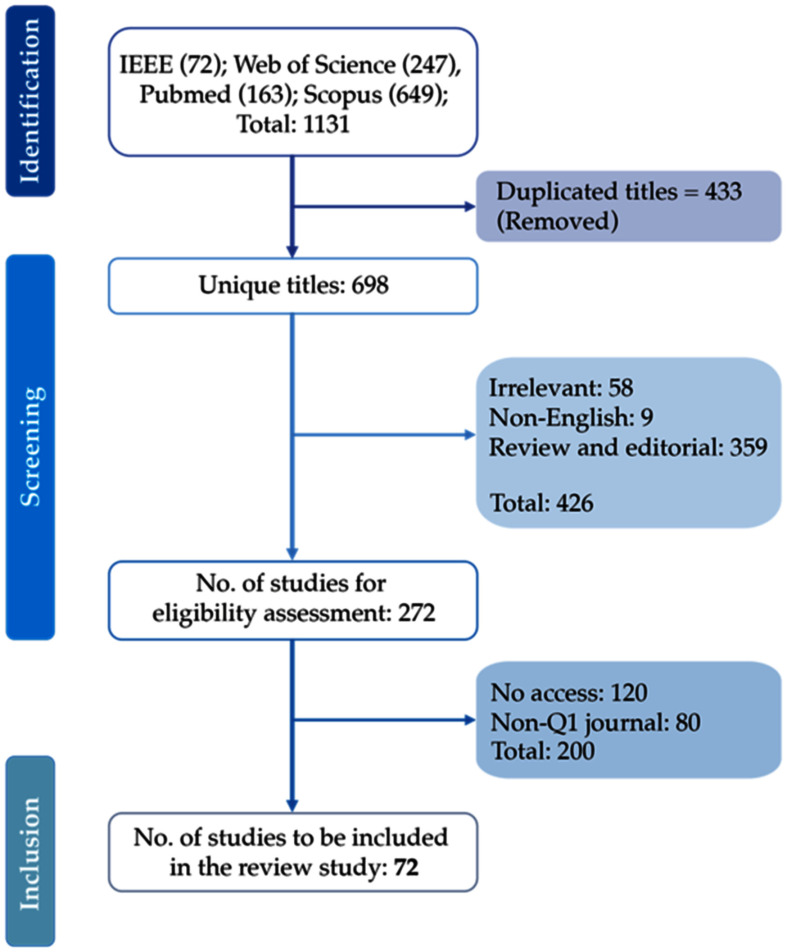
Flowchart of paper selection using PRISMA guideline.

Only top-quartile (Q1) open-access publications were retained. Final selection criteria are listed in Table I. This process resulted in 72 studies focusing on GAN applications in medical images and signal analysis. Fig. 2 illustrates the PRISMA article selection flowchart.

**TABLE I:** Inclusion and Exclusion Criteria

Inclusion criteria	Exclusion criteria
• Research focusing on the analysis, evaluation and processing of images obtained through medical imaging technologies (e.g., MRI, CT, etc.) • Studies were carried out using ECG, EEG, EMG, EOG, and PPG biomedical signals. • Research involving real patient data and using medical records obtained from this data. • Research aiming to increase the diversity and volume of existing medical datasets using GAN-based methods. • Studies that evaluate the research results based on specified performance metrics (such as PSNR, SSIM, accuracy, and F1 score) and report the results of at least one of these metrics.	• Studies involving medical records such as electronic health records and laboratory results. • Studies that do not specify the dataset used or that conduct research only with previously produced fake data. • Research that performs data augmentation using generation methods such as autoencoder. • Studies that conduct the research without providing any performance metrics and studies that evaluate only figures and whose numerical values are not specified.

## IV. Results

This section contains a detailed review of the examined studies, with each subheading corresponding to a medical GAN-based image and signal generation study of a specific human organ/part or clinical domain. The information contained in the reviewed studies is shown in Supplementary Material (Tables S1-S9).

### A. Brain Studies

GANs demonstrate potential in generating synthetic brain scans and simulating disease progression. Most of the 13 reviewed studies used magnetic resonance imaging (MRI), with some incorporating computed tomography (CT) and positron emission tomography (PET) images (Table S1). The focus was predominantly on brain tumors, using both public and private datasets. Original image counts ranged from 400 to 274000, with generated images varying from 512 to 200000 per GAN model.

Various GAN architectures were employed, including PGGAN, cGAN, Pix2Pix, and CycleGAN. Performance was evaluated using metrics like PSNR, SSIM, and Dice score. The highest reported PSNR (45.66) and SSIM (0.97) were achieved by Li et al.'s [24] Transformers-based GAN, while Khalil et al.'s [25] cGAN model obtained the best Dice score (95.2%).

### B. Oncology Studies

GANs in oncology address data scarcity and improve diagnostic accuracy. Studies use diverse imaging modalities, including MRI, CT, PET, mammography, cone beam CT (CBCT), and microscopy (Table S2). The limited availability of high-technology imaging methods underscores the need for synthetic data generated by GAN-based methods.

Original image counts ranged from 11 patients to 18084, with generated images spanning 15 to 75000. Various GAN architectures were employed, including cGAN, DCGAN, CycleGAN, Pix2Pix, and StyleGAN. Performance metrics included peak signal-to-noise ratio (PSNR), structural similarity index measure (SSIM), mean absolute error (MAE), and Fréchet inception distance (FID). Baydoun et al. [26] achieved the highest PSNR (63.41) and SSIM (0.98) values using a shallow U-Net cGAN.

### C. Lung and Breast Studies

GANs generate diverse synthetic mammograms and chest X-rays, potentially improving early detection algorithms. Of 8 studies, 7 focused on COVID-19 detection in lung X-ray and CT images (Table S3). The pandemic highlighted the need for large datasets to achieve high accuracy in AI-based diagnostics.

Reviewed studies aimed to develop non-invasive imaging methods for COVID-19 detection using GAN-based data augmentation. Original image counts ranged from 446 to over 1 million, with generated images varying from 1000 to 32440. GAN architectures included PGGAN, DCGAN, cGAN, CycleGAN, and StyleGAN. Qin et al. [27] achieved the best FID (152.73) and SSIM (0.43) values using PGGAN.

GAN-based data augmentation improved diagnostic performance. Waheed et al. [59] reported increased accuracy (85% to 95%), sensitivity (69% to 90%), and specificity (95% to 97%) for COVID-19 detection. Korkinof et al. [28] demonstrated a high similarity between PGGAN-generated and real mammography images.

### D. Ophthalmology Studies

GANs generate synthetic retinal images, aiding in the development of diagnostic tools for rare eye conditions. Five studies used private datasets, highlighting limited public data access (Table S4).

Studies focus on various imaging modalities, including color-coded corneal tomography, color fundus photography (CFP), and optical coherence tomography (OCT). Original image counts range from 561 to 276113, with generated images varying from 71 to 900000. GAN architectures include Pix2Pix cGAN, ProGAN, StyleGAN, PGGAN, StyleGAN2, and Pix2PixHD. Wang et al. [29] achieved the best SSIM (0.9508) and lowest FID (6.8) using StyleGAN2.

GAN-based data augmentation improved diagnostic performance. Sreejith Kumar et al. [30] reported an AUC of 0.90, a sensitivity of 86.7%, and a specificity of 69.4% for glaucoma detection using PGGAN. Kim et al. [31] demonstrated the realism of StyleGAN-generated images through an image Turing test.

### E. Hearth Studies

GAN-based methods help overcome limitations in heart-related datasets by increasing image numbers, reconstruction, and facilitating image-to-image translation (Table S5).

Studies focus on different imaging modalities, including MRI, ultrasound, and CT (Table S5). Original image counts range from 15 3D heart volumes to 1778, with generated images varying from 27 3D heart volumes to 1000. GAN architectures include CycleGAN and 3D Pix2Pix. Gilbert et al. [32] achieved the best Dice coefficients for real (0.94) and generated (0.87) images using CycleGAN.

GAN-based data augmentation shows potential for improving diagnostic models in cardiology. Amirrajab et al. [33] used SPADE-GAN to generate MRI images, achieving Dice coefficients comparable to real images for the left ventricle, right ventricle, and myocardium segmentation.

### F. Gastrointestinal Studies

GANs generate synthetic endoscopic images for training detection algorithms. Most studies focus on polyp segmentation and classification (Table S6). Two studies used endoscopic images, while one used X-ray images for gastritis classification.

Original image counts range from 815 to 107060, with generated images varying from 100 to 30000. GAN architectures include PGGAN and SinGAN. Togo et al. [34] used a combination of loss function-based conditional PGGAN and PGGAN for gastritis classification, achieving a sensitivity of 0.77 and specificity of 0.74 for generated images, compared to 0.87 and 0.88 for real images.

### G. Musculoskeletal Studies

GANs in musculoskeletal imaging generate synthetic X-ray or MRI images of bones and joints, focusing on leg muscles and knee images (Table S7).

Studies utilize ultrasound and MRI modalities. Original image counts range from 100 to 237883, with 5100 generated images reported. GAN architectures include CycleGAN, GAN, and HieGAN. Denck et al. [35] achieved NMSE of 0.09, PSNR of 24.48, and SSIM of 0.66 for MRI-contrast-aware image-to-image translations. Gan et al. [36] presented HieGAN, achieving MAE of 0.00135 and Wasserstein distance of 0.506 for synthetic MRI images.

### H. Other Image-Based Studies

This section covers studies using images from various organs and modalities, including kidney, abdominal, cell, dentistry, urology, hematology, head, histopathology, cytogenetics, and radiology (Table S8).

Various imaging modalities include MRI, CT, X-ray, microscopy, intraoral images, cyan fluorescent proteinC (CFP), and CBCT. Original image counts range from 60 patients to 354814 images, with generated images varying from 200 to 15540. GAN architectures include CycleGAN, cGAN, PGGAN, StyleGAN, and Pix2Pix.

Saleem et al. [37] achieved high performance in generating microscopy images for hematology, while Müller-Franzes et al. [38] reported low FID scores for histopathology images using StyleGAN3.

### I. Signal-Based Studies

This section focuses on studies using biomedical signals such as ECG, electroencephalogram (EEG), electromyography (EMG), electrooculography (EOG), and photoplethysmography (PPG) (Table S9). Most studies were conducted in cardiology, some involving neurology and clinical microbiology. The primary objectives were classification or segmentation, using GAN-based methods to improve accuracy, sensitivity, and specificity. GAN models typically consisted of 1D Convolutional Neural Network (CNN)-based generators and discriminators.

Original signal counts range from 48 records to 110000 ECG beats, with generated signals varying from 8000 to 21837 records. GAN architectures include SynSigGAN, GAN, cGAN, ProGAN, and DC-Wasserstein GAN. Hazra and Byun [39] achieved low FID and root mean squared error (RMSE) values for EEG, PPG, and EMG signals using SynSigGAN. Alcaraz et al. [40] reported high AUC values for generated ECG records.

## V. Discussion

### A. Overview of GAN Applications in Medical Imaging and Signal Analysis

GAN models offer numerous advantages in healthcare, including data augmentation, anonymization, quality improvement, modality transformation, artificial data generation, disease simulation, and multi-task learning. This review examines 72 publications on synthetic data generation in healthcare using GAN models across seventeen clinical fields.

**Fig. 3.**
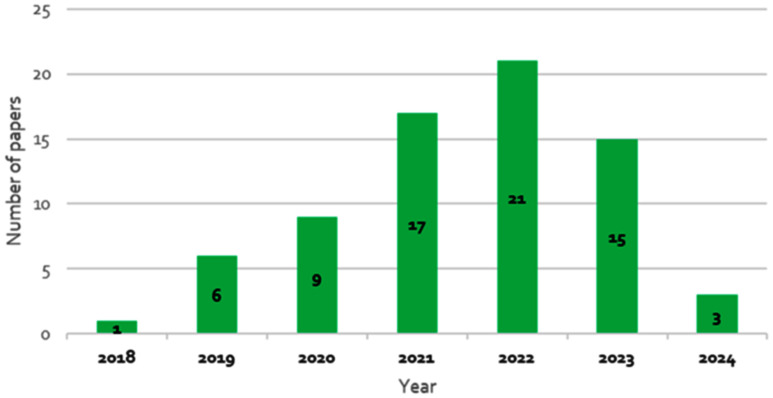
Distribution of published articles by year (2018–2024) showing the growing adoption of GANs in healthcare. The significant increase since 2019 reflects both technological maturity and increasing recognition of GANs' potential in medical applications.

**Fig. 4.**
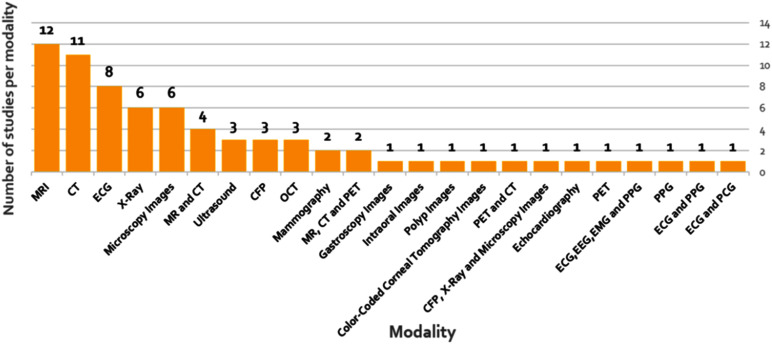
Distribution of studies by imaging modality. MRI and CT emerge as dominant modalities, followed by ECG signals. This distribution highlights areas where GAN applications have gained traction and identifies potential opportunities in underrepresented modalities.

As illustrated in Fig. 3, the number of research articles investigating GAN-based approaches in healthcare has gained significant momentum since 2019, indicating a growing interest and acceptance of these techniques in the medical research community. As shown in Fig. 4, MRI and CT techniques dominate the studies, followed by synthetic ECG generation. Brain studies constitute the most researched clinical domain, followed by biomedical signals, cancer, ophthalmology, and lung-related research. The prevalence of MRI-based studies can be attributed to data accessibility and MRI's capability to provide detailed soft tissue images. ECG-based studies are prominent due to lower acquisition costs and widespread use in cardiac health assessment.

**Fig. 5.**
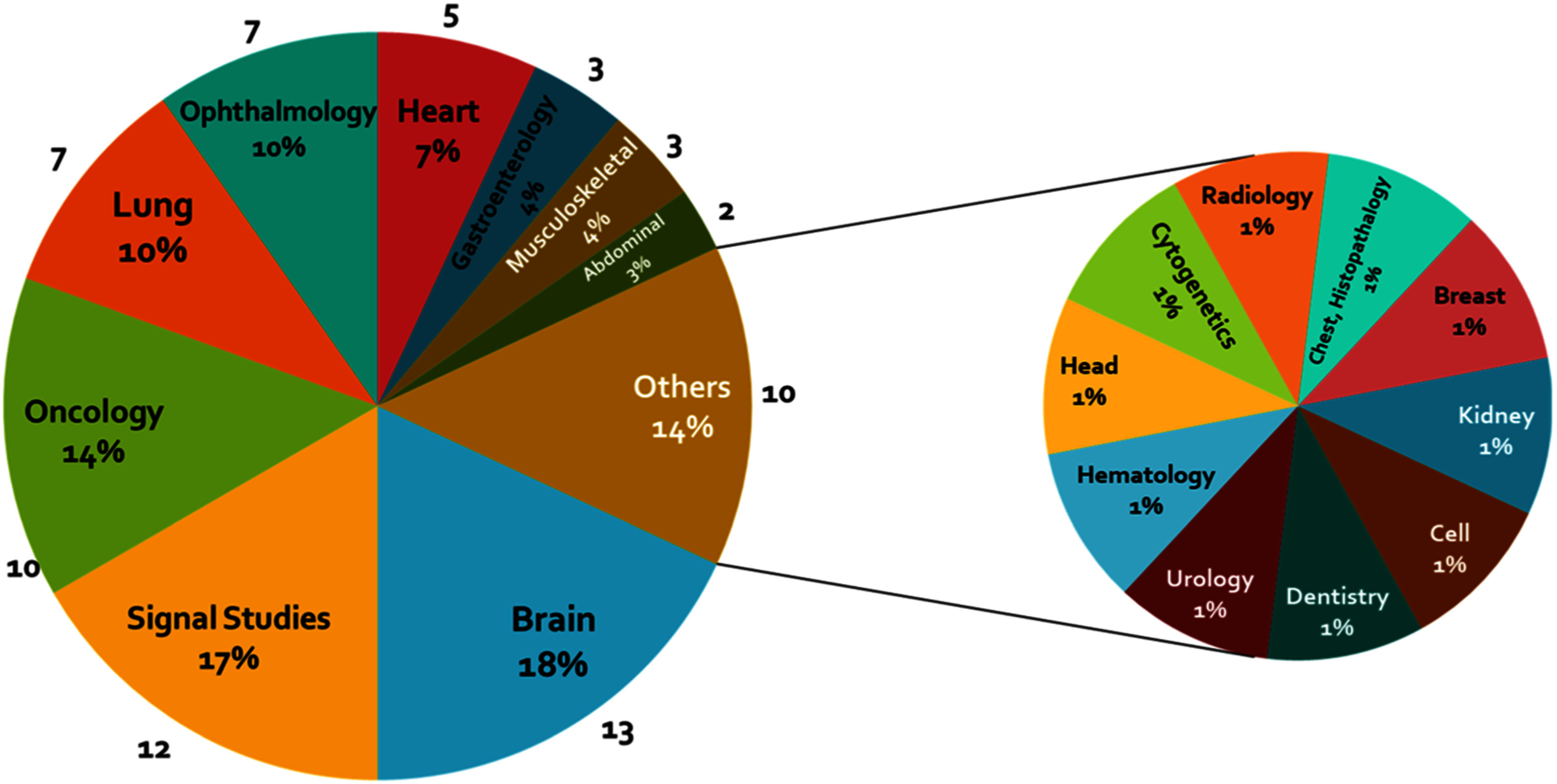
Distribution of studies across clinical domains. The chart reveals brain studies (18%) and signal studies (17%) as leading areas, demonstrating GAN's utility in these domains while also highlighting potential gaps in other medical fields.

While GAN applications span various clinical domains, some areas like hematology, cytogenetics, and urology remain underexplored. The disparity between image-based and signal-based studies suggests potential for further research in biomedical signal processing using GAN-based methods (Fig. 5).

### B. GAN Architecture for Synthetic Data Generation in Healthcare

**Fig. 6.**
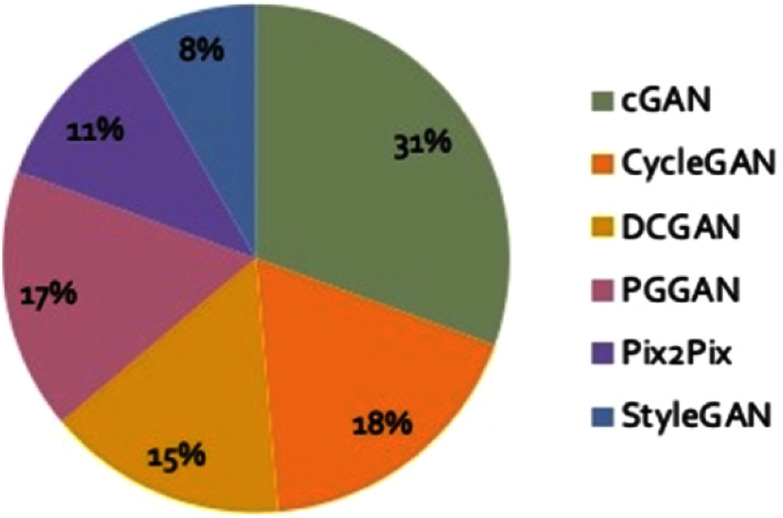
Pie-of-pie chart of GAN architectures employed in the reviewed studies, where cGAN (31%) and CycleGAN (18%) represent the most common choices. This distribution reflects the evolution of GAN implementations to meet various healthcare challenges.

The review reveals increasing interest in GAN applications for healthcare data generation (Fig. 6). Table II shows that cGAN was the most frequently used architecture (31%), particularly in brain, cancer, and ophthalmology applications, due to its ability to incorporate conditional information. CycleGAN (18%) was second, especially in brain and lung studies, favored for its unpaired data learning capability.

**TABLE II:** Most Common GAN Architectures Based on the Task Performed

Task	GAN architecture
Data augmentation (n= 33)	cGAN (n=12), DCGAN (n= 8), PGGAN (n=5), StyleGAN (n=4), CycleGAN (n=2), Pix2pix (n= 2)
Domain translation (n= 26)	CycleGAN (n= 8), cGAN (n= 6), Pix2pix (n=6), PGGAN (n=3), DCGAN (n=2), StyleGAN (n=1)
Data enhancement (n= 13)	cGAN (n= 4), CycleGAN (n=4), PGGAN (n=4), DCGAN (n=1), StyleGAN (n=1), Pix2pix (n= 0)

The analysis of the 72 reviewed papers reveals architecture-specific strengths and limitations across clinical domains. Conditional GANs demonstrated superior performance in brain imaging applications, achieving SSIM values between 0.82-0.97 [24], [25], particularly excelling in paired image translation tasks. However, their performance showed notable degradation (SSIM: 0.66-0.75) in applications requiring fine structural preservation [41]. CycleGAN architectures proved particularly effective in unpaired translation scenarios, achieving impressive results in cardiac imaging with Dice coefficients ranging from 0.87 to 0.94 [32], though they consistently struggled with preserving minute anatomical details in high-resolution scenarios. StyleGAN variants have shown exceptional capability in ophthalmology applications, achieving remarkable performance metrics (FID: 6.8, SSIM: 0.95) [29], particularly for high-fidelity retinal image synthesis, albeit requiring substantial computational resources.

### C. Limitations of This Review

Key limitations include the lack of standardization in performance metrics, which complicates direct comparisons between studies. The absence of clearly specified numbers of synthetic images produced in many studies and inconsistent reporting of image quality assessment metrics (e.g., PSNR, SSIM, FID) further hinder comprehensive analysis. Challenges arise in comparing studies using different methods and datasets. In signal-based studies, inconsistent data properties and meta-parameters complicate comparisons. The use of varied performance metrics across studies makes synthesis difficult.

Despite these limitations, the review establishes the current landscape of GAN-based medical data generation, highlighting progress and remaining challenges.

### D. Challenges of GANs in Healthcare

The application of GANs in healthcare presents several challenges. Technical challenges specific to GAN architectures significantly impact healthcare applications. Mode collapse, where generators produce limited varieties of samples, particularly affects rare disease representation and limits the diversity of synthetic medical data [42]. Training instability manifests through oscillating losses and failing convergence, especially in medical imaging where fine pathological features must be preserved.

The adversarial training process, involving simultaneous optimization of generator and discriminator networks, introduces additional complexity compared to traditional deep learning approaches. Computational demands present another barrier, as high-performing implementations typically require specialized GPU resources [29].

Quality assessment poses another significant challenge. Traditional metrics often fail to capture synthetic image quality, leading to reliance on alternative metrics that may miss artifacts identifiable by human experts. Studies utilizing smaller datasets (fewer than 1000 samples) showed average SSIM reductions of 15–20% compared to those with larger datasets [26], highlighting the critical impact of data quality and quantity. The lack of robust metrics for evaluating GAN training progress in medical contexts further complicates model selection and optimization.

Domain-specific challenges have also emerged. In brain imaging, studies reported difficulties in preserving small lesions and fine pathological features [43], [44], while cardiac imaging applications may struggle with temporal consistency [32], [33]. In data augmentation scenarios, limited diversity in generated samples remains a significant issue, especially with scarce training data. GANs may produce samples with low diversity when trained on small or homogeneous datasets, potentially compromising the utility of synthetic data for tasks such as rare disease simulation or enhancing model robustness.

### E. Opportunities and Future Directions

GANs offer opportunities to advance medical decision support systems. Our analysis reveals several critical areas for development. Data simulation for training operators represents a promising direction, as GANs can generate realistic medical data to train professionals, improving diagnostic skills [45]. This is particularly valuable in rare disease scenarios or for training in resource-limited settings.

While emerging technologies show promise, GANs maintain distinct advantages in specific scenarios, particularly with limited datasets. GANs perform better in resource-constrained scenarios, as seen by recent fundus photography generation, which shows improved picture quality (FID score: 41.761) and much shorter training durations (30 hours versus 250 hours for diffusion models) with minimal datasets [46]. These benefits become more important in specialized medical fields where computational resources may be constrained, or huge datasets may not be available. Transfer learning approaches present another promising direction for enhancing GAN performance in healthcare. Recent advances in transfer learning for medical image analysis have demonstrated significant improvements in model efficiency and accuracy [47], [48], [49].

The development of standardized performance metrics within specific clinical areas is a crucial need. Current evaluation frameworks show inconsistency across studies, and clinical significance is not well captured by conventional measures like SSIM and PSNR. The creation of no-reference metrics is crucial for assessing GAN-based techniques in the absence of ground truth data [50], since they may be used as integrated loss functions to enhance model training.

Technical innovations should focus on some promising approaches to the generative models identified in our review. Data standardization using GANs shows promise in dermatology [51] and digital pathology [52], ensuring consistent analysis across different settings [53]. Architecture enhancement should also prioritize the development of lightweight variants optimized for clinical deployment, particularly addressing the computational efficiency-performance trade-off observed in current implementations.

Enhancing the interpretability and explainability of GAN-based solutions becomes essential for safe clinical integration [54], especially in critical diagnostic applications.

Recent advancements in generative AI, including diffusion models and large language models like GPT-4 and BERT [55], show promise in healthcare applications. While diffusion models have demonstrated impressive results in medical imaging [56], [57], particularly for unconditional image generation, both GANs and diffusion models have complementary strengths in different clinical scenarios. Future studies should adopt hybrid approaches that combine GANs with diffusion models or transformers to leverage the strengths of each technology and address their respective limitations.

GANs remain particularly effective for applications requiring paired data translation, real-time processing, or high-resolution image generation, while typically demanding fewer computational resources. The coexistence of multiple generative approaches highlights the importance of selecting appropriate tools based on specific clinical needs [24]. However, challenges remain regarding transparency [58], [59], ethical considerations, and potential biases. Future developments should focus on creating norms and regulations for fair, responsive, and reliable implementation of these technologies in healthcare [60], balancing potential benefits with responsible development and use.

## VI. Conclusion

This systematic review provides a comprehensive overview of synthetic data generation via GANs in healthcare. Our analysis shows these models have been incorporated in various applications across several clinical domains, with an increasing presence in brain, signal, cancer, ophthalmology, and lung studies. Applications include generating synthetic data, enhancing image quality, augmentation, anonymization, and multi-task learning. While there is promising evidence of progress in GAN-based approaches in healthcare, challenges remain, including the absence of agreed-upon metrics for cross-study comparisons. This aspect is further complicated by the domain-specific nature of medical data quality assessment. Our analysis identifies promising avenues for further research, including the development of reference-free metrics to evaluate generated data quality and improved methods for preserving fine pathological features. The coexistence of GANs with emerging technologies like diffusion models suggests a future where multiple generative approaches complement each other, chosen based on specific clinical requirements. New applications of GANs, including training medical workers and simulating realistic clinical scenarios, present exciting opportunities for future exploration, as long as development prioritizes both technical advancement and practical clinical utility.

## Author Contributions

**Muhammed Halil Akpinar**: Methodology; Formal Analysis; Data Curation. **Abdulkadir Sengur**: Formal Analysis; Writing - Original Draft. **Massimo Salvi**: Methodology; Writing - Original Draft; Visualization. **Silvia Seoni**: Validation; Data Curation; Visualization. **Oliver Faust**: Writing - Review & Editing. **Hasan Mir**: Writing - Review & Editing. **Filippo Molinari**: Supervision; Writing - Review & Editing. **U. Rajendra Acharya**: Conceptualization; Supervision; Writing - Review & Editing. All authors contributed to the article and approved the submitted version.

## Conflict of Interest

The authors declare that they have no conflicts of interest.
